# Surface enhanced Raman spectroscopy and machine learning as an accurate and rapid diagnostic tool for hydrocephalus

**DOI:** 10.1038/s41598-025-32177-6

**Published:** 2025-12-16

**Authors:** Jorge Servert Lerdo De Tejada, Derren J. Heyes, Shumaila Chaudhry, Shahid Iqbal, Naima Mehdi, Danylo Komisar, Oleksii Ilchenko, Jaleel A. Miyan

**Affiliations:** 1 Division of Neuroscience, Faculty of Biology, Medicine & Health, The University of Manchester, Manchester, UK; 2 Manchester Institute of Biotechnology, Faculty of Science & Engineering, The University of Manchester, Manchester, UK; 3 Departments of Neonatology, The Children’s Hospital Lahore, University of Child Health Sciences, Lahore, Pakistan; 4 Departments of Neurosurgery, The Children’s Hospital Lahore, University of Child Health Sciences, Lahore, Pakistan; 5 Departments of Microbiology, The Children’s Hospital Lahore, University of Child Health Sciences, Lahore, Pakistan; 6 Lightnovo ApS, Birkerød, Denmark

**Keywords:** Biological techniques, Nanoscience and technology, Neurology, Neuroscience

## Abstract

Hydrocephalus is a severe neurological disorder marked by cerebrospinal fluid (CSF) accumulation in the brain’s ventricles, causing elevated intracranial pressure, neurological deficits, and substantial societal and medical costs. Rapid detection in neonates and children remains challenging, as primary diagnostic indicators such as increased head size often manifest late, delaying intervention. Hydrocephalus stems from diverse causes, including prematurity, intracranial bleeds, CNS infections, and brain tumours. CSF collected for diagnostic purposes provides an opportunity to explore innovative rapid detection methods. In this paper, we show that integrating Surface-Enhanced Raman Spectroscopy (SERS) and machine learning offers a novel molecular diagnostic tool for hydrocephalus. Using silver nanoparticle-layered-cellulose strips as SERS substrates and a portable Raman spectrometer, we analysed CSF samples from 117 patients (70 controls, 47 hydrocephalus cases). Within 5 min of sample placement in strip, the optimized Random Forest algorithm achieved 97% accuracy in blind testing, with 100% specificity and 95% sensitivity. The miniaturized Raman spectrometer and standarised strips enable portability and support clinical use, particularly in resource-limited settings. Our grid search-based ML workflow and scoring system enable the prediction of overfitting, as well as assessment of feature importance within the spectra, improving model interpretability, which could prove helpful in other vibrational spectroscopy applications. Overall, this study provides evidence of an accurate, rapid, portable, and interpretable diagnostic technique for hydrocephalus. Further validation with larger cohorts will refine predictive models and expand clinical utility, advancing diagnostic precision for hydrocephalus and related neurological conditions.

**Supplementary Information:**

The online version contains supplementary material available at 10.1038/s41598-025-32177-6.

## Introduction

 Hydrocephalus is a major neurological condition affecting 1:500 live human births globally, 1:700 in the USA,1:1000 in Europe and 1:100 in many developing countries in Africa, Asia and South America^1–3^with a high neurosurgical burden^1,4^. It is characterized by accumulation of cerebrospinal fluid (CSF) within the ventricles and/or external fluid spaces around the brain with associated raised intracranial pressure and brain damage if not treated^5^. Later-onset hydrocephalus can result in serious conditions including epilepsy and dementia^6,7^. Many metabolic disorders can lead to hydrocephalus with early diagnosis critical to prevent brain damage and significant effects on development^8–11^. CSF is secreted into the ventricles by the choroid plexus as well as having contributions from interstitial fluid passing into the CSF from the brain parenchyma^12^. Approximately 25 ml is held within the ventricles and a further 100 ml fills the subarachnoid space around the brain and spinal cord. Under normal conditions, approx. 500 ml enters the ventricular CSF space every day although this is modulated by various conditions affecting the choroid plexus or interstitial fluid production as well as by osmotic changes mediated by macromolecule deposition^13^. Drainage/removal of CSF from the cranium thus becomes a critical aspect for brain health and function. This occurs across the arachnoid membrane where active transport of CSF via pinocytosis transports CSF across the membrane to drain into venous sinuses^14^. There is also drainage into facial and dural lymphatics as well as proposed routes out of the head along peri-vascular and peri-neural pathways^15,16^. Many of these pathways are low pressure pathways with the arachnoid activated by higher pressure pulses and sustained pressure^14^. Over production of CSF is another cause of hydrocephalus usually associated with choroid plexus tumours and certain inflammatory conditions that upregulate aquaporins to increase interstitial fluid and consequently increase CSF volume. Obstruction of CSF flow or clearance/drainage pathways commonly result in hydrocephalus, enlargement of the ventricles and compression of the brain with associated neurological sequelae. The degree of ventricular enlargement is determined by the degree of obstruction to CSF flow and/or drainage and thus presents in a spectrum of disorders associated with various degrees of fluid accumulation^7,12,17^. Fluid accumulation outside the ventricles, in the subarachnoid spaces is classified as benign external hydrocephalus as it has no association with neurological problems except where accumulation continues to increase. Recently, this condition has been associated in children who develop autism spectrum disorder^18^.

Congenital and foetal acquired hydrocephalus are usually obvious before or at birth due to the large head size. Postnatal hydrocephalus due to birth asphyxia, infection or other trauma is less easy to diagnose as it can develop slowly appearing like normal head size increases at first. Head size increase can be gradual or accelerating but both need urgent treatment to prevent irreversible brain damage and death. Hydrocephalus charities have recently endorsed the “get -a-head” initiative for neonatal and child head size measurements to capture hydrocephalus in early stages (https://harrys-hat.org/get-a-head/), however this remains voluntary and a hit and miss approach to early detection. In our previous studies, we have found a profound change in CSF composition and metabolic profile in hydrocephalus focused on cerebral folate metabolism^9,19,20^ but with multiple pathways affected^19^. Indeed, a major change in CSF composition associated with fetal-onset hydrocephalus in the hydrocephalic (Texas) rat model of congenital hydrocephalus was observed that indicates major changes in brain metabolism and physiology associated with this condition and underlying the developmental arrest we have found^19–21^. The developmental arrest and hydrocephalus could be prevented and/or reversed using simple folate supplements^21^ that further highlights the need for early diagnosis. Overall, these findings indicate that analysing the biochemical composition of lumbar-puncture-extracted CSF could support diagnosis, provided it is conducted using a technique that is both accurate and rapid.

Spontaneous Raman spectroscopy is a non-destructive analytical method that measures the inelastic scattering of monochromatic light as it interacts with molecular vibrations to determine chemical composition^22^. This method makes it possible to identify and describe materials according to their spectral fingerprints, which enable reliable identification of complex molecules^22^. Surface-enhanced Raman spectroscopy (SERS) can enhance Raman signals by between 10^6^ and 10^15^ times by amplifying the Raman scattering signals of molecules adjacent to nanostructured metal surfaces, such as gold or silver nanoparticles^23^. This amplification produces a localized surface plasmon resonance magnifying the Raman signal of nearby molecules. This occurs by the plasmonic effect, by which the electrons in the nanoparticles coherently oscillate due to the laser’s electromagnetic field. SERS is especially well-suited for identifying low-concentration biomarkers in liquid biopsies because of its increased sensitivity^23^. An example of this is the diagnosis of colorectal cancer through serum analysis with a 77% sensitivity and an 83% specificity^23^, prostate cancer in urine with a 95% accuracy rate^24^, and Sjogren’s syndrome in saliva with a 94% accuracy rate^25^. CSF analysis using SERS has enabled Alzheimer’s disease prognosis through β-Amyloid quantification^26^, highlighting its use for neurological disorders.

Machine learning (ML) plays a crucial role in interpreting complex spectral data from SERS, allowing the distinction of clinical features in high-dimensional, noisy datasets^27^. While deep learning models are popular for medical diagnosis, their “black-box” nature often limits interpretability, validation, and regulatory approval^28^. To overcome this, dimensionality reduction techniques like Principal Component Analysis (PCA) and Partial Least Squares (PLS) regression are commonly employed to simplify the data, making it more interpretable by identifying the most important spectral features^29^. These methods work well in combination with classification algorithms like linear discriminant analysis (LDA), quadratic discriminant analysis (QDA), and support vector machines (SVM), all of which generate decision boundaries that are easier to interpret. In contrast to dimensionality reduction^29^, feature selection techniques like Random Forest (RF) focus on identifying the most relevant regions in the spectral range directly, excluding redundant regions^29,30^. RF is an ensemble learning method that builds multiple decision trees using random subsets of features. It classifies data by aggregating the predictions of all trees, using majority voting for classification. RF ranks features based on their ability to reduce model error, automatically identifying the most important features while discarding less relevant ones, which improves model stability, accuracy, and interpretability, making it ideal for medical diagnostics^30^.

To enhance clinical translation, our methodology prioritizes minimal sample pre-processing. In this study, silver nanoparticle-layered-cellulose strips were used as SERS substrates to analyse 117 pediatric cerebrospinal fluid (CSF) samples—70 controls and 47 from hydrocephalus patients—collected from three distinct medical centres. The strips were selected for their strong signal enhancement properties and ease of use, while the LightNovo-ApS MiniRaman spectrometer (785 nm) provided a portable and affordable alternative to traditional Raman setups, facilitating clinical applicability. Following data collection, grid search optimization^31^ was applied to tune hyperparameters across multiple classification models (LDA, QDA, SVM, and RF). We performed stratified 10-fold cross-validation on stratified 70% of the data to select the best model through our scoring system. In contrast to traditional metrics (such as the Akaike and Bayesian Information Criteria or raw k-fold accuracy, which can be skewed by outlier folds, class imbalance, or noise memorization^32^, we adopt a portfolio-theory–inspired scoring approach^33^ that jointly rewards class-balanced performance and penalizes overfitting. Within each fold we compute a Weighted Balanced Accuracy (WBA) that treats minority classes equally and summarize these via the median (MWBA). We then apply an explicit overfitting penalty based on the gap between training and validation accuracy (the full scoring formula is presented in Sect. 4.5). This method prioritizes models that perform consistently across folds, treat all classes fairly, and generalize robustly^34^. To avoid data leakage and overfitting, we tested these selected models in the remaining, unseen, stratified 30% of the data, obtaining the final statistical metrics. The RF model, with 2,000 trees, a maximum tree depth of 2, a minimum of 30 samples required to split an internal node, 15 samples required at each leaf, and a ‘log2’ feature-subsampling strategy, achieved the best results, yielding a testing accuracy of 97.22%, 95.45% specificity, and 100% sensitivityl, demonstrating its robustness in diagnosing hydrocephalus. The diagnostic process took a total of 5 min since the sample was placed in the strip: 2 min to allow the sample to spread through the cellulose, 3 min to take the measurements under the Raman spectroscometer, and (once trained) less than 1 s (0.37 s) for the RF to classify the sample. By integrating portable, affordable technology with machine learning techniques, this study establishes a strong foundation for future predictive and classification models aimed at improving the diagnosis and management of hydrocephalus.

## Results

### CSF fingerprinting in hydrocephalus and control patients using SERS

**Fig. 1:**
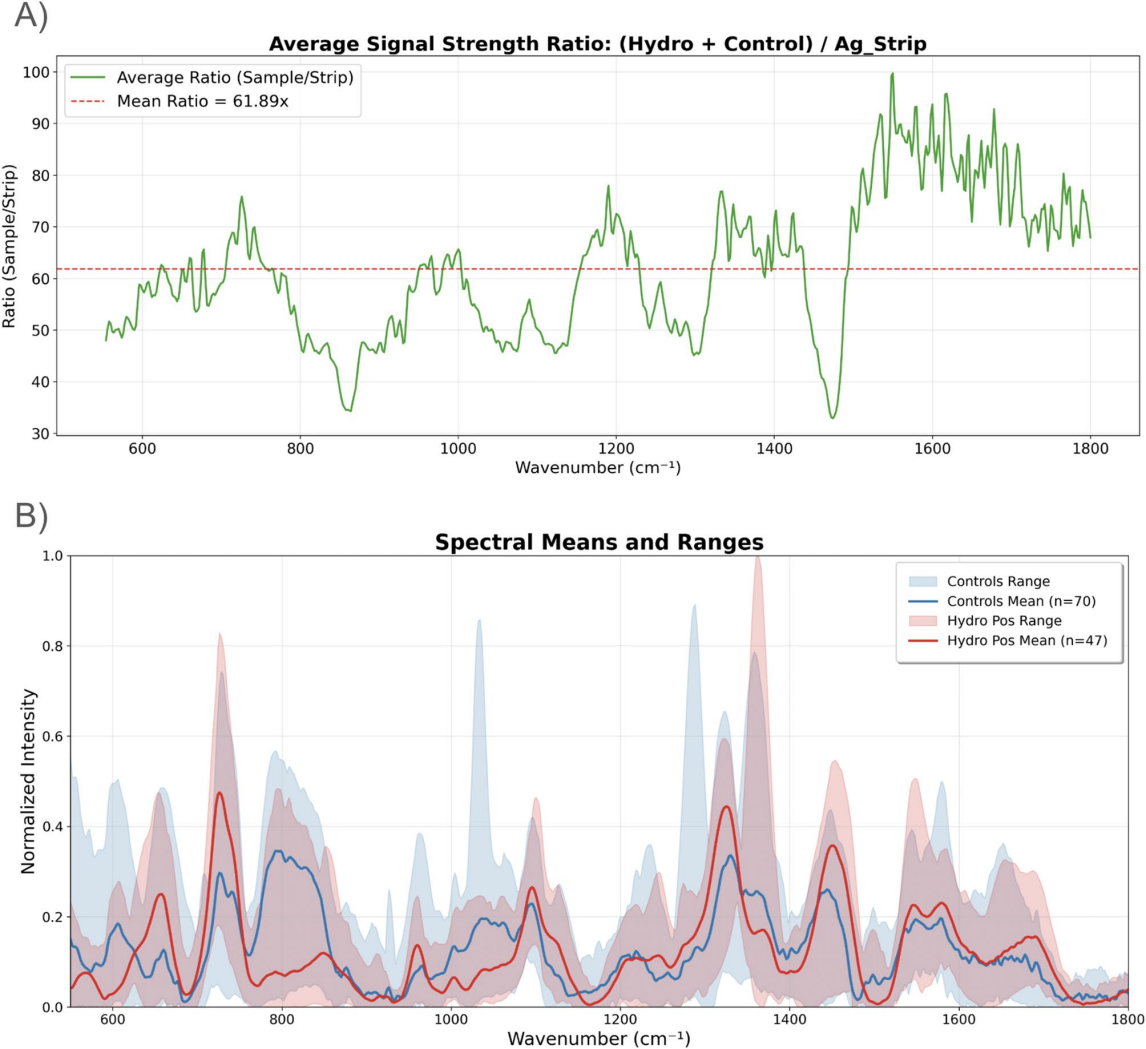
SERS fingerprinting analysis of CSF from hydrocephalus and control patients. (**A**) Average signal stregnth ratio between sample spectra (hydrocephalus + control) and mean AgNP strip background, plotted across the Raman shift range. The green trace shows the average intensity ratio across the Raman range, and the dashed red line indicates the overall mean ratio (61.89×), confirming minimal contribution from the AgNP Strip. (**B**) Mean baseline corrected and vector normalized SERS spectra (solid lines) and spectral intensity ranges (shaded areas) for hydrocephalus (red) and control (blue) samples.

The MetroHM Silver P-SERS substrates (Ag-NP strips) were assessed for repeatability and reproducibility by collecting five Raman measurements, at different positions in the Ag-NP-coated area, from ten strips. Coefficients of variation (CVs) were calculated within each strip and across strips, both before and after vector normalization. Mean CVs were 1.16% (repeatability) and 5.44% (reproducibility) before normalization, improving to 1.50% and 2.08% afterward (Details around these measurements can be found in Supplementary Materials 1). These values outperform typical SERS substrates, which often show 5–25% RSD, and the ~ 2% post-normalization reproducibility approaches that of highly controlled laboratory substrates^35^. This demonstrates adequate structural uniformity, thus being chosen for this study. Furthermore, the Raman intensity from the strips alone was, on average, 61.89-fold lower than after CSF was added (Fig. 1A), ensuring minimal feature interference or diagnostic bias.

To evaluate the variations in their SERS profiles, CSF samples from 118 patients (Controls *n* = 70, hydrocephalus *n* = 48) were subjected to SERS analysis using the AgNPs strips. Controls have a slightly lower mean CV (60.28%) compared to hydrocephalus (65.02%), with relative ranges of 265.12% and 390.20%, respectively. The reason for these variations can be explained by the diversity of CSF composition^7,9,12,17,19–21^, as well as the complex associative behaviour of silver nanoparticles in biofluids, with slight variations in protein, metabolite and even anionic content affecting the aggregation of biomolecules to the nanoparticles^36,37^. Figure 1B presents the total range (Supplementary Materials 2 presents the collection of spectra) of both groups against the mean after substraction of the Ag-NP strip signal (Details about the substraction process and effects in the signals can be seen in Supplementary Materials 3). The high variance in the spectral features prompted our use of dimensionality reduction, denoising, and machine learning techniques to improve classification accuracy.

The comparison of mean Raman spectra between the control and hydrocephalus groups reveals several key spectral differences. Notably, the control group shows higher intensities at 600 cm⁻¹, 790 cm⁻¹, and 1350 cm⁻¹, which are associated with vibrations from lipids, proteins, and C–C stretching^38,39^. The 900 cm⁻¹ and 930 cm⁻¹ peak, related to C–H bending vibrations^40^, appear to only be present in control CSF. The 710 cm⁻¹ and 1320 cm⁻¹ peak, associated with C–C stretching^22^, and the peaks around the 1540 cm⁻¹ and 1690 cm⁻¹ region, corresponding to amide I bands (C = O stretching)^22,41^, pose a higher intensity in the hydrocephalus CSF compared to controls. All these differences highlight potential biochemical distinctions in the CSF of Hydrocephalus patients, which follows observations published in the literature^7,9,12,17,19,20^. Further analysis with methods such as Mass Spectrometry (MS) and Nuclear Magnetic Resonance (NMR) could provide a better explanation for the nature of these differences.

### Dimensionality reduction using PCA, PLS and RF

**Fig. 2:**
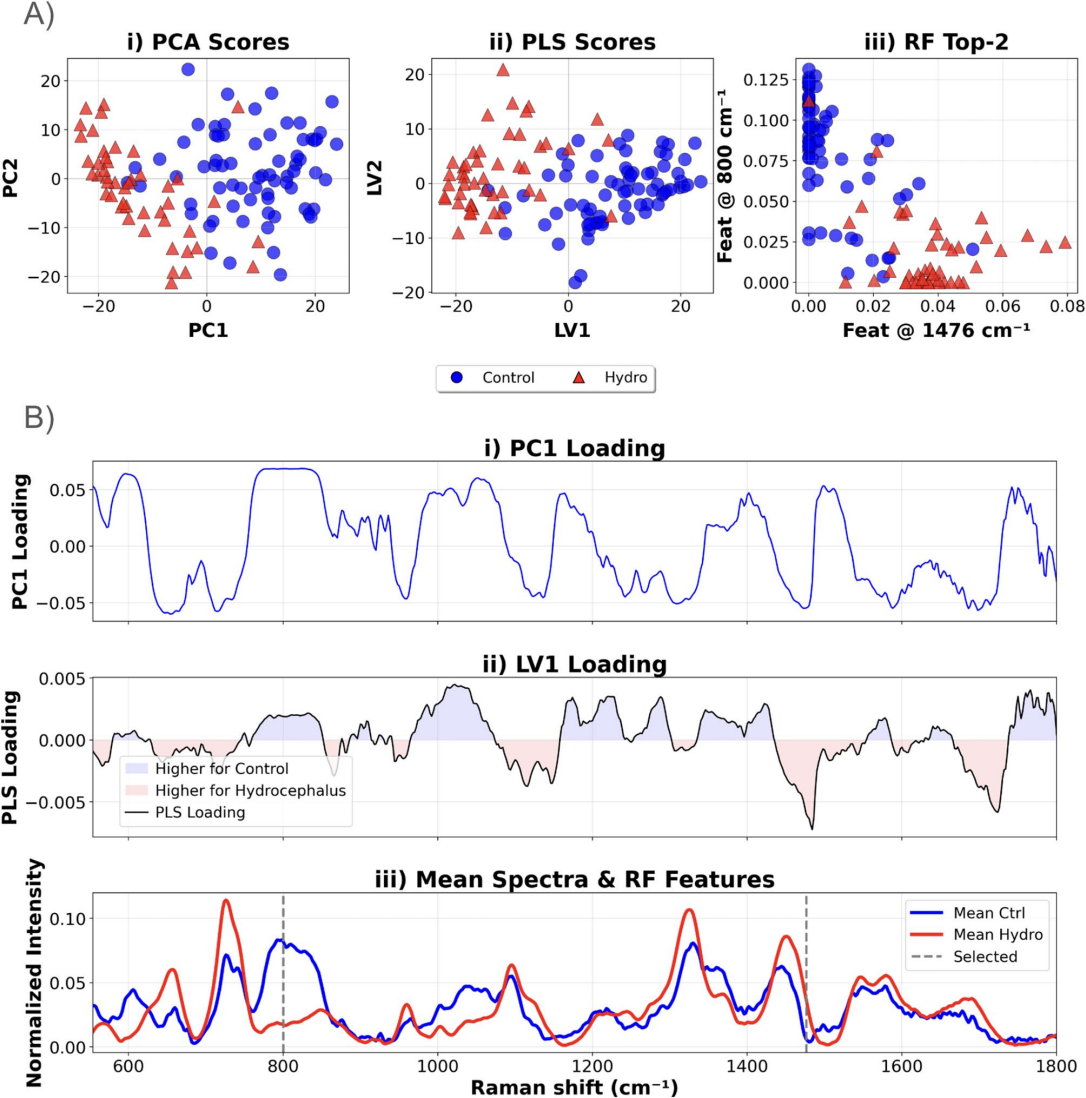
Dimensionality reduction: Multivariate analysis of normalized Raman spectra from control (blue) and hydrocephalus (red) samples Panel **A**(i) shows a PCA scatter plot of PC2 versus PC1; **A**(ii) displays a PLS scatter plot of LV2 versus LV1; and **A**(iii) presents a scatter plot of the two top-ranked RF features at 800 cm⁻¹ (y-axis) and 1476 cm⁻¹ (x-axis). Panel **B**(i) plots the PC1 loading profile across all wavenumbers; **B**(ii) overlays the LV1 PLS loading curve with red shading indicating bands more intense in hydrocephalus and blue for those stronger in controls; and **B**(iii) compares the mean normalized spectra of each group, with dashed vertical lines highlighting the RF-selected bands at 800 and 1476 cm⁻¹.

We utilized PCA, PLS, and RF to reduce the dimensionality of the spectral data, exclude noise and identify significant patterns. PCA extracts a set of orthogonal variables based on the variance of the data (X-variance)^29^, enabling dimensionality reduction in an unsupervised manner. Due to the variance in the data, 22 PCs were required to explain 95% of the variance, with the first 3 components covering 52% of the data. Figure 2A(i) presents the scattered plot of the PC1 (29% explained variance) and PC2 (13% explained variance). The shape of the PC1 loading plot, (Fig. 2B(i)), shows the contribution of different spectral regions to the component, presenting the directionality provided by each peak. These areas coincide with mean differences between both groups as observed in Fig. 1B. PLS optimizes the covariance between predictors (X) and response variables (Y), enabling supervised dimensionality reduction^29^. Figure 2A(ii) shows the separability between the control (blue circles) and hydrocephalus (red triangles) groups across LV1 in the 2D scatter plot, as it presents a high Fisher ratio of 0.86. Nevertheless, separability appears to fall significantly in LV2, with a Fisher ratio of 0.53 compared to 2.35 in LV1. Figure 2B(ii) shows similarities with the PC1 loading curve, with the feature directionality being highlighted in blue and red.

In addition to PCA and PLS, RF was employed as a tool for feature selection, enabling a different approach to dimensionality reduction. RF works by constructing multiple decision trees and using the majority vote to classify the data^29,30^. The RF embeds feature selection by identifying the most relevant features by evaluating the “importance” of each Raman shift region, allowing to pinpoint key spectral bands associated with hydrocephalus (red triangles) and control (blue circles) groups. In our analysis, shifts around 800⁻¹ and 1476 cm⁻¹ were identified as the main features for differentiation. These selected features were visually highlighted in the mean spectra plot (Fig. 2B(iii)). In the 2D scatter plot of the top 2 features selected through RF (Fig. 2A(iii)), the points show a clearer clustering compared to PCA and PLS.

### Classification model selection and testing accuracy

**Fig. 3:**
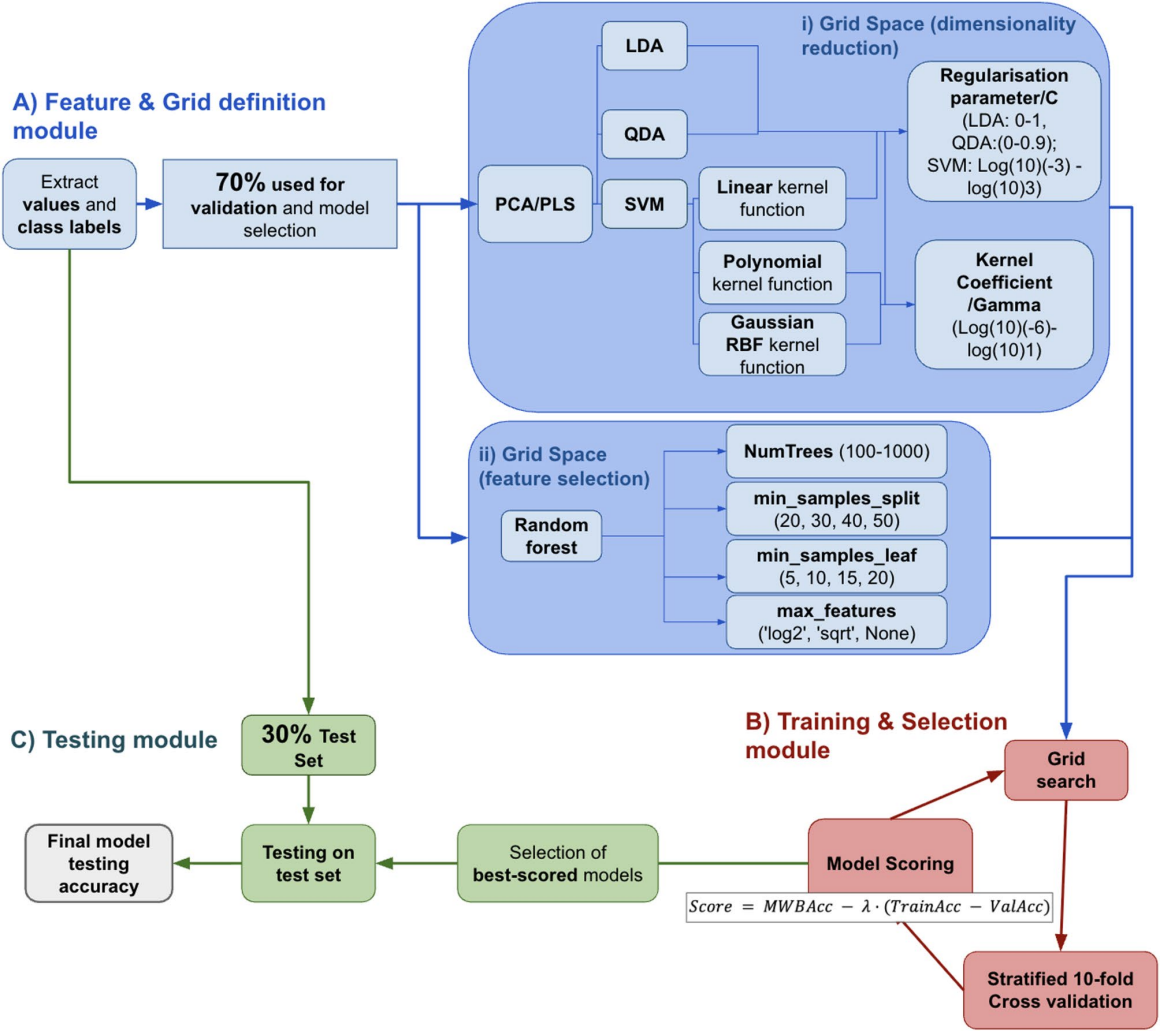
Workflow of the model selection and validation process: (**A**) Feature & Grid Definition Module: The dataset is split into a stratified 70/30 train-test set, with 30% of the data reserved as an unseen set for final testing. The training set undergoes dimensionality reduction using PCA/PLS and feature selection via RF, as well as hyperparameter range setting. (**B**) Validation & Selection Module: Hyperparameters and dimensions are fine-tuned through grid search with stratified 10-fold cross-validation, selecting the combinations that provide the best score. (**C**) Testing Module: The final models are tested on the unseen set to determine its accuracy and generalizability.

The flow diagram in Fig. 3 outlines the algorithm used to develop and select the best diagnostic approach by comparing different dimensionality reduction and classification methods. In the Feature and Grid Definition module (Fig. 3A, blue), the Raman spectral values and class labels were extracted, and the data was split into two sets in a stratified manner: 30% for testing and 70% for training and validation. Different dimensionality reduction (PCA/PLS) and feature selection (RF) methods were compared with this training and validation set. Various classification methods using PCA/PLS, including LDA, QDA, and SVM, were tested with different Regularisation/C and Kernel Coefficient/Gamma values, and a grid search was performed to find the best combinations (Fig. 3A(i)), Grid Space (Dimensionality Reduction)). RF was also optimized by tuning the number of trees, minimum samples per split and leaf, and feature selection strategy via grid search (Fig. 3A(ii)), Grid Space (Feature selection)). Each algorithm was scored using our customary scoring system, favoring high MWBA from stratified 10-fold cross-validation and penalizing overfitting, measured by the difference between training and validation accuracy across the learning curve. Finally, in the Testing Module (Fig. 3C, green) the selected models were tested on the unseen 30% dataset to evaluate its final performance.

**Fig. 4:**
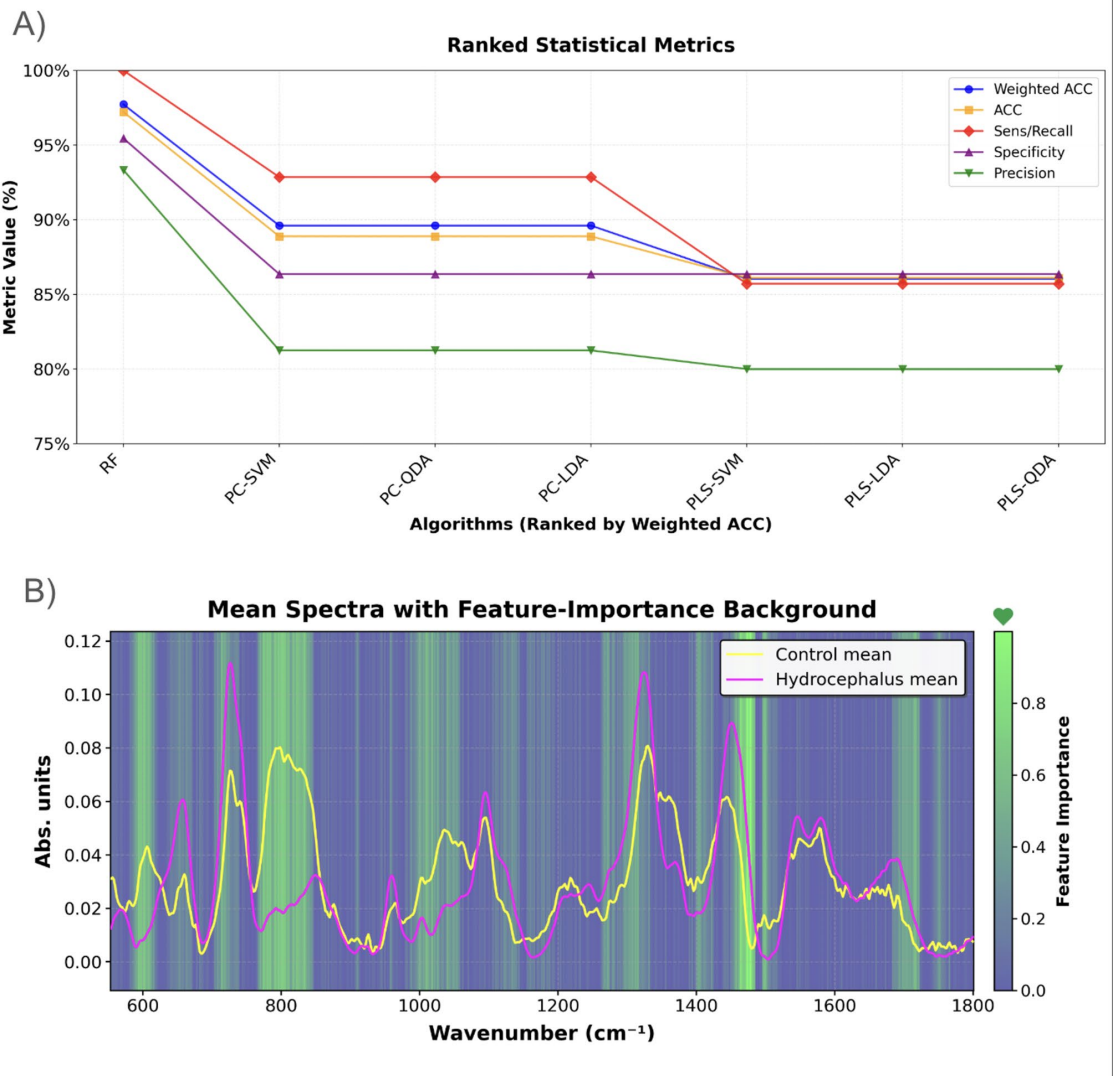
Classification Performance Metrics and Spectral Feature Importance (**A**) Ranked statistical performance metrics for the seven classification algorithms. Algorithms are ordered by weighted bakanced accuracy, with each curve representing a different evaluation metric: weighted accuracy, overall accuracy, sensitivity/recall, specificity, and precision. Random Forest achieves the highest overall performance, followed by PC-SVM, while the remaining models show lower but comparable performance across metrics. (**B**) Mean Raman spectra for control and hydrocephalus groups overlaid on a feature-importance heatmap derived from the RF classification model. Yellow and magenta lines represent group-averaged spectra, while the background color scale indicates the relative importance of spectral wavenumbers for model discrimination (Greener for stronger discrimination, blue for weaker discrimination).

Following the selection process, each model’s performance against the testing split was assessed based on their WBA, accuracy, sensitivity, and specificity. Precision and recall were included in the analysis to assess the algorithm’s bias in detecting hydrocephalus patients^21,42^. This was crucial to address the 42% data imbalance favoring the controls and to ensure the model remained highly sensitive, which is essential for diagnosing a life-threatening condition. RF (2,000 trees, a maximum tree depth of 2, a minimum of 30 samples required to split an internal node, 15 samples required at each leaf, and a ‘log2’ feature-subsampling strategy) performed significantly better than the other methods, with a WBA of 97.73%, 97.22% accuracy, 95.45% specificity, 93.33% precision, and 100% sensitivity/recall. Once trained the RF algorithm took 0.37 s to classify samples. The runner-up was PCA-SVM, with a with 89.61% WBA, 88.89% accuracy, 86.36% specificity, 81.25% precision, and 92.86% sensitivity/recall. Supplementary Material 4 offers numerical information about the performance of all other models, as well as additional assessments for the RF algorithm such as ROC and learning curve. The difference in performance appears to be mostly driven by the dimensionality reduction technique, rather than the discrimination algorithm, as can be seen by the comparability of the performance across all PCA and PLS methods. Figure 4 A offers a visual comparison of these statistical metrics across models.

Figure 4B overlays the normalized mean spectra for control (yellow) and hydrocephalus (magenta) samples on a green–blue heatmap of feature importance from the final RF, where each wavenumber’s importance reflects its contribution to reducing node impurity across all trees^30,43^. The strongest importance bands appear around 600 cm⁻¹, 710–720 cm⁻¹, 750–780 cm⁻¹, 1000–1075 cm⁻¹, 1250–1300 cm⁻¹, 1470–1500 cm⁻¹, and 1700–1750 cm⁻¹each coinciding with clear divergences between the two group means. These regions exhibit both sharp peaks and broad shoulders in importance, indicating that the classifier relies not only on isolated discriminative features but also on extended spectral intervals carrying cumulative biochemical differences, while the widespread mid-level importance across the baseline reflects the RF’s ability to aggregate numerous subtle signals and thereby improve overall robustness.

## Discussion

This study demonstrates the potential of combining SERS with machine learning for the diagnosis of hydrocephalus. Our method uses minimal sample pre-processing, accessible materials, and interpretable classification models. By leveraging RF for dimensionality reduction and classification, we have provided an accurate and interpretable diagnostic tool that can identify hydrocephalus-specific alterations in CSF composition/metabolism, yielding a diagnostic accuracy of 97.22%. The model’s 100% sensitivity with a minimal specificity compromise (95.45%) makes it a robust method to detect a life-threatening condition like hydrocephalus. We also believe that the grid search-based ML workflow presented in this paper, as well as our scoring system, could be employed for the diagnosis of other conditions^24,27^ and other fields here vibrational spectroscopy interpretation is required, such as the biopharmaceutical industry^44,45^.

This idea came from our previous findings of significant changes in CSF composition and cerebral metabolism in congenital and neonatal hydrocephalus^9,19–21^ in which we found an almost complete change in composition and multiple metabolic pathway changes orchestrated by the central folate metabolic cycle and the important folate enzyme 10-formyl tetrahydrofolate dehydrogenase (FDH, aka ALDH1L1). In hydrocephalus, FDH is significantly reduced or absent from CSF triggering the major metabolic changes that underlie the block in DNA synthesis and cortical development^19,21^. A sensitive analytical technique, such as SERS in our case, could therefore enable broader diagnoses using CSF as an analyte. The significant nature of the physiological changes in CSF composition and their effect on global cerebral metabolism make CSF an ideal candidate for Raman spectroscopy and diagnosis of this devastating neurological condition. Our studies indicate that these changes in composition occur very early in the aetiology of hydrocephalus^9,19–21^, which could make our approach an early detection technique. However, prospective longitudinal cohort study with paired (within-subject) sampling is required to verify this. Early diagnosis and treatment would prevent significant loss of brain development due to cerebral folate blockade, as well as preventing damage to brain tissue due to fluid accumulation and increasing intracranial pressure. These factors are the major causes of severe neurological outcomes where treatment is late^46,47^. Moreover, our recent research demonstrates similar changes in CSF metabolic profiles associated with enlargement of the cerebral ventricles without hydrocephalus, a hallmark feature associated with increasing severity of many cerebral neurological conditions^7^. It seems possible, if not probable that our diagnostic method could be applicable to any conditions where ventricular enlargement is occurring due to the metabolic changes that occur.

The current study used 117 samples of neonatal CSF from 70 control and 47 affected individuals. Clearly, the method would be greatly improved and validated with a larger cohort of patients, and we are currently collecting samples from multiple centres with high incidence of this condition. The learning curve provided in Supplementary Materials 4 shows that the model achieves high accuracy, and validation performance continues to improve even at the largest training sizes. This suggests the model is robust but still data-limited, meaning additional samples would likely push performance even higher. With the much larger cohort, it may also be possible to distinguish the different causes and aetiologies of hydrocephalus and apply more specific treatments to prevent the negative neurological outcomes. We know already, for example, that for congenital hydrocephalus a simple treatment with natural folates, but not folic acid can prevent and/or reverse the hydrocephalus^21^. We are currently acquiring samples for this purpose.

The SERS silver nanoparticle strips provide a translatable platform to carry out the Raman analysis with minimal sample processing. It is entirely possible that the use of other nanoparticles with alternative morphologies (e.g., rods or stars)^48^or larger ensembles of composite structures may provide higher intensities and higher reproducibility via optimisation of hotspot overlaps or electromagnetic fields through inter-nanoparticle distance^49,50^. Composite structures such as core–shell or 2D “gap-controlled” configurations lock in these hotspots, while reducing uncontrolled aggregation^49–51^. Moreover, gold and bimetallic nanoparticles are alternative options to improve chemical stability and biocompatibility^52^. In addition, the integration of mapping strategies like motorised stages for sample mapping^53^ or Rotating/scanned laser illumination^54^ could significantly improve coverage, increasing repeatability and reproducibility. Finally, combining the SERS analysis with complementary analytical techniques, such as MS and NMR, or labelling nanoparticles with molecular tags (e.g., antibodies or nucleotide strands) could improve diagnostic accuracy further^55^.

In conclusion, this study demonstrates the usefulness of a rapid and interpretable approach to hydrocephalus diagnosis using SERS and machine learning. By employing RF, we achieved high diagnostic accuracy while preserving model transparency, providing an alternative to conventional diagnostic methods. As the field of clinical SERS advances, this approach holds promise not only for hydrocephalus but also for other neurological disorders where accessible, accurate, and interpretable diagnostics are needed. Future research focusing on substrate standardization, model generalization, and validation with complementary techniques will be essential to translate these findings into a clinically viable diagnostic tool.

## Materials and methods

### Clinical approval and sample collection

All methods were performed in accordance with the relevant guidelines and regulations from The University of Manchester and all the institutions involved in the study. All experiments were performed after approval from The University of Manchester Research Ethics Committee, as well Institutional Research Ethics Committee of the University of Child Health Sciences-Children’s Hospital Lahore. Informed consent was obtained from the parents/guardians of all the participants. NHS (Newcastle) Research Ethics committees approved UK Hospital sample collections. Samples from a total of 117 patients (control *n* = 70, hydrocephalus *n* = 47) were analysed (Supplementary Materials 5 provides further sample details). Samples were collected from Lahore Children’s Hospital (control *n* = 70, hydrocephalus = 27), Leeds General Infirmary (hydrocephalus *n* = 8), and Alder Hey Children’s Hospital (hydrocephalus *n* = 12). CSF extraction by clinicians followed standard lumbar puncture or ventricular tab procedures after parental consent was obtained for use of excess CSF to clinical diagnostic in this research. The excess CSF was labelled by clinicians and placed in a -80 °C freezer within one hour of extraction. The samples were then transported to the University of Manchester under the same constant temperature conditions, along with their corresponding anonymized laboratory results and clinical observations.

### Sample preparation and SERS substrate for hydrocephalus diagnosis

One of the main goals of this study was to assess the possibility of diagnosing hydrocephalus by employing minimal sample pre-processing, ensuring minimal hurdles related to laboratory devices and trained personnel. For this reason, the CSF samples were simply centrifuged to minimize signal contributions from debris, after which the supernatant was placed in a separate tube, pipetted up and down to ensure homogeneity, and aliquoted into 50 µL volumes. All samples were subjected to a maximum of one freeze-thaw cycle before measurement to preserve CSF integrity^56^. After thawing, 25 µL were pipetted onto silver nanoparticle-layered-cellulose strips onto chromatography cellulose strip (MetroHM’s Silver P-SERS Substrates). Supplementary material 6 presents a morphological characterisation of the nanoparticle distribution over the cellulose strip. The sample was allowed to spread through the cellulose for 2 min, then placed over an aluminium foil-covered glass slide, to enhance reflectivity^57^, and analyzed by Raman spectroscopy using a 785 nm laser.

### Raman measurement protocols and data pre-processing

A MiniRaman spectrometer (LightNovo, SERS 785 nm model) with a focusing stage were used to ensure consistent laser spot size and signal across samples (Supplementary Material 7 offers visual of the set-up). The 785 nm laser wavelength was chosen to minimize any interference from background fluorescence signals^57^. Before taking measurements, the Raman spectrometer was calibrated using a silicon standard, with a distinct band observed around 520 cm⁻¹. Each SERS strip was positioned under the laser (50 μm spot size) and the device was vertically adjusted for optimal signal acquisition. Miraspec software was used to control the measurement parameters that were set to a 10 s exposure time, 20 gain, and 10 mW power. For the characterisation of the MetroHM Silver P-SERS substrates (Ag–NP strips), we measured 5 Raman spectra at different locations on each of 10 strips to quantify repeatability (within-strip CV) and reproducibility (across-strip CV). CVs were calculated before and after vector normalization. We performed a sum of 18 accumulations per strip, totalling 3 min measurements. For the strips that included the CSF, we followed the same 3 min measurement procedure, taking the measurement in the same position on the strip to obtain a single representative spectrum per sample.

Spectra were preprocessed using a custom Python (version 3.11.4) module that standardized all files before analysis. TSV spectra were first loaded and cropped to 550–1800 cm⁻¹. Substrate contributions were corrected by subtracting the mean AgNP_Strip reference prior to any normalisation or baseline correction (using the raw measured signal) (As seen in Suplementary Material 3). After reference removal, sample spectra underwent asymmetric least-squares baseline correction (λ = 1 × 10⁴, *p* = 0.001, 10 iterations), ensuring non-negative corrected values. Finally, all spectra were scaled to unit length using L2 vector normalization.

### Dimensionality reduction and feature selection techniques: PCA, PLS, and RF

To improve classification accuracy and maximise interpretability, this study assessed two main avenues: dimensionality reduction through PCA and PLS, and feature selection though RF. PCA and PLS reduce data dimensionality by projecting it onto a smaller set of principal components (PCs) or latent variables (LVs), capturing the most variance^29^. PCA focuses on variance in the numeric variables (X-variance) and PLS on the categorical variables (Y-variance)^29^. In contrast, RF directly selects the most important features for classification, potentially improving both model performance and interpretability^29^.

PCA was used to retrieve the principal components from the predictor matrix using Python’s compute_pca function. The cumulative variance captured by the components could be examined using this function, which returns each component’s scores, loadings, and explained variance. Based on the variance explained, a score matrix was generated to guide the subsequent grid search, where the classificatory algorithms assessed 1–10 dimensions as the input.

To increase the covariance between the predictor matrix and class labels during the dimensionality reduction step, Python was employed to perform the PLS transformations and return scores and loadings. Specifically, the PLSRegression function was applied to the predictor. The number of PLS dimensions was iterated from 1 to 10 to explore the influence of PLS components on classification accuracy in the following grid search algorithms.

Random Forest modelling trained on all available features without performing any feature selection beforehand. The parameter *max_features=’log2’* specified that, at each decision-tree split, the algorithm should randomly sample a subset of features whose size equals the base-2 logarithm of the total number of input features. This sampling step is a built-in mechanism that introduces randomness and decorrelation between trees, but it does not remove any features from the overall model. After training, feature importance was computed using the mean decrease in impurity to quantify the contribution of each wavenumber; however, these importance values were used only for interpretation and visualization rather than for filtering or restricting the feature set during model construction.

### Classification model development and hyperparameter optimization

As mentioned before, the Raman spectral data were pre-processed in Python by separating the feature values, which represent the intensities of each Raman shift, and the class labels, where Control = 0 and Hydrocephalus = 1. The dataset was then divided in a stratified into a training-validation set (70%) and a virgin test set (30%) to prevent data leakage. This division was performed in a patient basis, meaning than a single spectrum from each patient was used as an observation for the classification process. Therefore 48 and 33 observations from controls and hydrocephalus were used for training, and 22 and 14 observations from controls and hydrocephalus were used for testing.

During training, for each candidate model we computed a single Score (Eq I) by taking its MWBA (Eq II) and subtracting an over fitting penalty λ × (TrainAcc – ValAcc). MWBA is defined as the median of the K fold wise weighted balanced accuracies WBA (Eq III) which guards against any single fold dragging the result. Within each fold WBA is the average of true positive rate and true negative rate but class weighted by ω_i_ (Eq IV) where each class contribution is scaled inversely to its sample count so that small classes count just as much as large ones. The penalty term comes from inspecting learning curves (plots of training accuracy versus validation accuracy at our fixed training size). A large vertical gap between those curves signals over fitting because the model is memorizing noise instead of learning general patterns. By explicitly subtracting that gap we steer hyper parameter selection toward models whose training and validation curves remain close together ensuring true generalization rather than just a high in sample score. This system of equations was defined as:

1 $$\:Score = MWBAcc - \lambda \cdot (TrainAcc - ValAcc)$$

2 $$\:MWBA=WBA\left(\frac{k+1}{2}\right),\mathrm{\:if\:}k\mathrm{\:is\:odd\:};\:\frac{WBA\left(\frac{k}{2}\right)+WBA\left(\frac{k}{2}+1\right)}{2},\mathrm{\:if\:}k\mathrm{\:is\:even\:}$$

3 $$\:WBA={\sum\:}_{i=1}^{k}{{\upomega\:}}^{i}\cdot\:\frac{1}{2}\left(\frac{T{P}^{i}}{T{P}^{i}+F{N}^{i}}+\frac{T{N}^{i}}{T{N}^{i}+F{P}^{i}}\right)$$

4 $$\:\omega\:i=\frac{1/Ni}{{\sum\:}_{j=1}^{k}\left(1/Nj\right)}$$

Here, Score (Eq I) is our final selection metric obtained by taking the multi weighted balanced accuracy and subtracting λ times the difference between training accuracy and validation accuracy; λ is the penalty weight (set to 2). TrainAcc and ValAcc are the mean accuracies on the training and validation splits, respectively, and k is the number of cross validation folds (ten in our case). MWBA (Eq II) is the median of the k fold balanced-accuracy values, and each fold’s balanced accuracy (WBA^I^, Eq III) is computed as the average of its sensitivity (TP^I^/(TP^I^+FN^I^)) and specificity (TN^I^/(TN^I^+FP^I^)), with each class’s contribution scaled by a weight ω^I^. That weight (Eq IV) is proportional to 1/N^I^, where N^I^ is the total sample count in fold i, so that larger folds carry more influence when determining the weighted median.

Hyperparameter search ranges were defined for model training, with the regularization parameter (C) for LDA and linear Kernel SVM varying from 0 to 1 in steps of 0.25, and for QDA the regularization parameter range was set to 0-0.9 (with 0.1 step, 1 was not used as that would linearise the QDA and effectively make it an LDA)^58^. Non-linear SVM models had their C parameter set between log10(-3) and log10(3) and Gamma between log10(-6) and log10(1), where C controls the trade-off between fitting the model closely and maintaining generalization, and Gamma defines the influence of each training point on the decision boundary^59^. The three kernel functions evaluated were the Linear Kernel (for linearly separable data), the Polynomial Kernel (degree 3) for capturing non-linear relationships, and the Radial Basis Function (RBF) Kernel, which transforms data into an infinite-dimensional space using a Gaussian function to model complex decision boundaries^60^. Random Forest models were optimized through grid search over the hyperparameters *NumTrees* (100–1000), *min_samples_split* (20, 30, 40, 50), *min_samples_leaf* (5, 10, 15, 20), and *max_features* (‘log2’, ‘sqrt’, None)^43,61^. Random Forest hyperparameters control how trees grow and how much randomness is injected into the ensemble: *NumTrees* sets the number of trees, *min_samples_split* and *min_samples_leaf* regulate the minimum data required to create internal nodes and leaves (controlling tree depth and overfitting), and *max_features* determines how many features are randomly sampled at each split to promote diversity among trees^43,61^. The best hyperparameter combination was automatically chosen by comparing the score in each model after 10-fold cross-validation (Supplementary Marerial 8 offers a visualisation of the process). The selected model was then tested against the virgin test set, enabling measurement of the model’s accuracy, sensitivity, specificity, precision, and recall^42^.

## Supplementary Information

Below is the link to the electronic supplementary material.

Supplementary Material 1

Supplementary Material 2

## Data Availability

Data is provided within the manuscript and supplementary files. All raw data pertaining to the publication can be obtained from the corresponding authors.
