# Supplementary material for: Surface enhanced Raman spectroscopy and machine learning as an accurate and rapid diagnostic tool for hydrocephalus
Source: Sci Rep. 2025 Dec 16;16:2659. doi: 10.1038/s41598-025-32177-6 (PMC12824180; doi:10.1038/s41598-025-32177-6)
Supplement: Supplementary file 1 — Supplementary Material 1 [file 41598_2025_32177_MOESM1_ESM.docx]

**SUPLEMENTARY MATERIAL 1 – AgNP Strip Characterisation (Repeatability and reproducibility)**

**
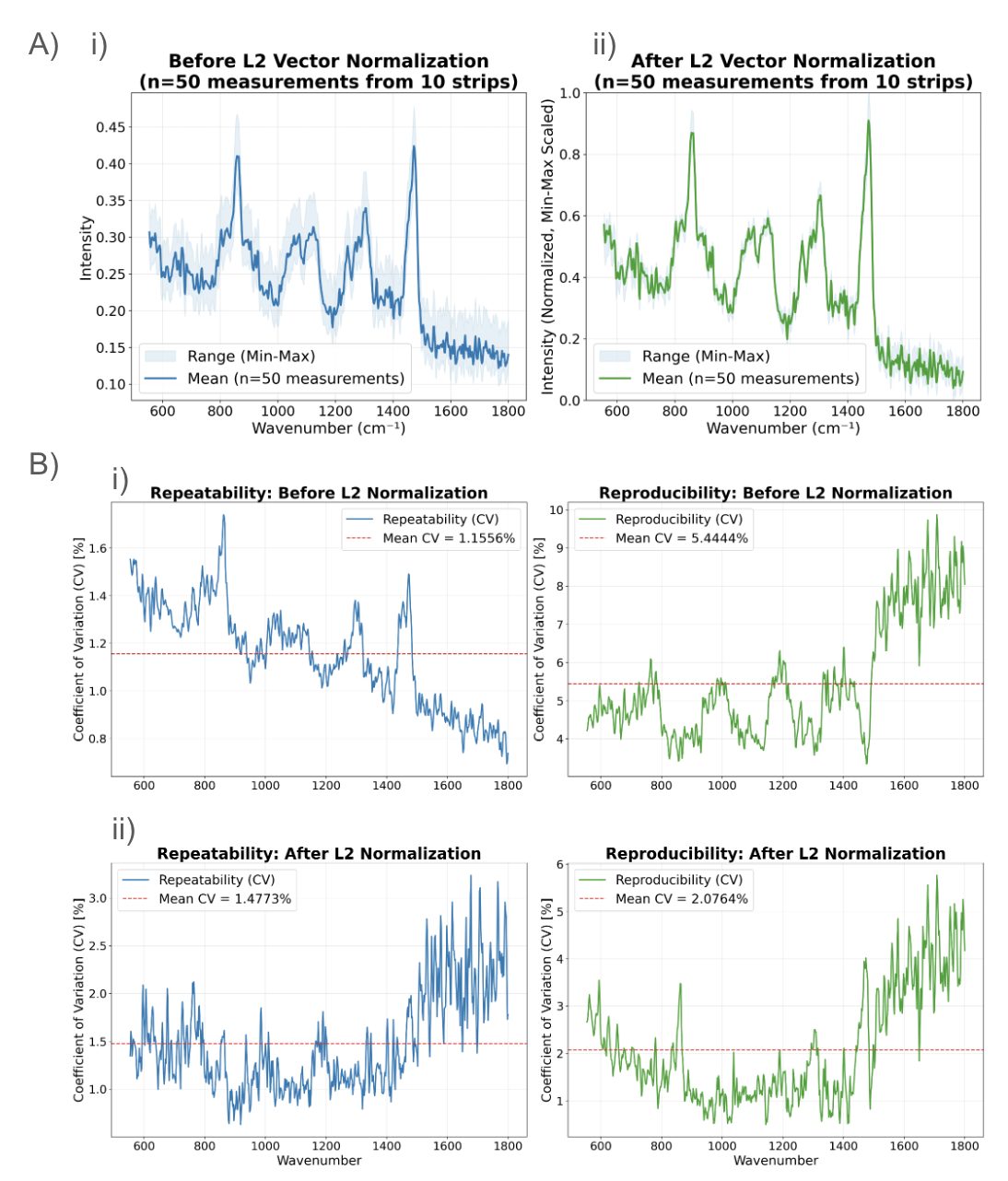
**

**Figure S.1 - Repeatability and reproducibility analysis on theAgNP strips.** Each strip was measured 5 times at different positions on the AgNP-coated side (10 strips total; n = 50 spectra). A) Mean spectra (solid lines) with min–max envelopes (shaded) before (i) and after (ii) L2 vector normalization across 554–1800 cm⁻¹. Normalization preserves spectral shape while reducing overall intensity variability across measurements. B) Coefficient of variation (CV) profiles showing (i) repeatability (within-strip variation) and (ii) reproducibility (between-strip variation). Before normalization, the mean repeatability CV was 1.1556% and the mean reproducibility CV was 5.4444%. After normalization, the mean repeatability CV was 1.4773%, while the mean reproducibility CV improved to 2.0764%. Horizontal dashed lines indicate the mean CV in each condition.

**SUPLEMENTARY MATERIAL 2 – Table with All Spectra Used in the Study.**

This information is in the file:

**Supplementary_Material_2_Table_With_All_Spectra.xlsx**

**SUPLEMENTARY MATERIAL 3 – Effect of AgNP Strip signal subtraction on the measured CSF spectra.**

**
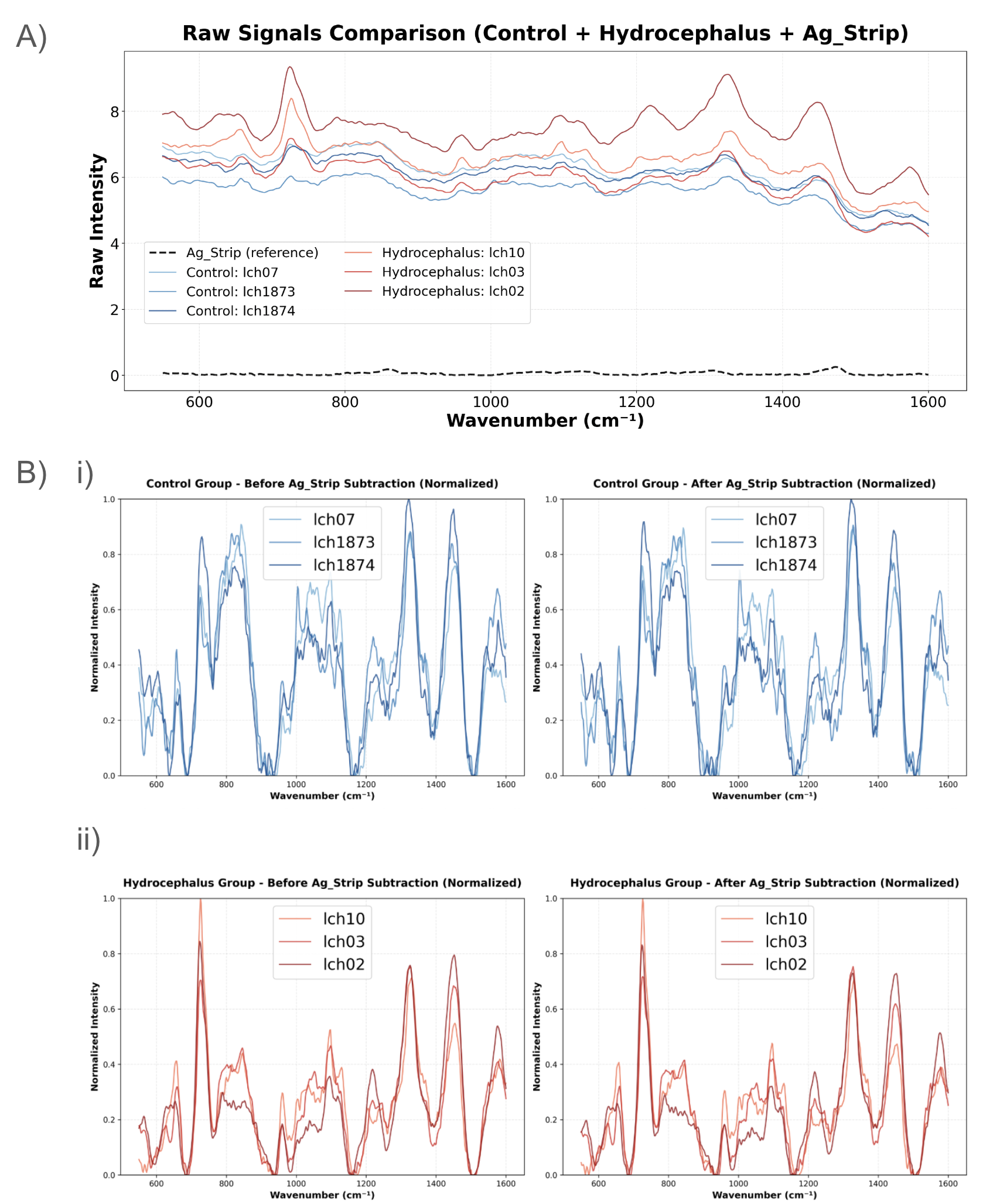
**

**Figure S.3 - Effect of AgNP Strip subtraction on representative control and hydrocephalus spectra.** A) Raw, unprocessed spectra from three representative control samples (Ich07, Ich1873, Ich1874) and three hydrocephalus samples (Ich10, Ich03, Ich02) compared against the AgNP_Strip reference spectrum. The CSF spectra show substantially higher intensity and clearer signal than the underlying AgNP_Strip, illustrating that the sample contribution dominates the measurement. B) Normalized spectra for the same control (i) and hydrocephalus (ii) samples before (left) and after (right) AgNP_Strip subtraction. The Cohen’s d was -0.01 for both groups, and the p value from the t test was 0.45 for hydrocephalus and 0.66 for controls, making the change in signal insignificant. Subtraction of the AgNP_Strip reference produces only minor adjustments to spectral shape, indicating that the strip contribution is minimal.

**SUPLEMENTARY MATERIAL 4 – Testing performance of the assessed models**

| **Algorithm** | **Weighted ACC** | **ACC** | **Sensitivity** | **Specificity** | **Precision** | **ROC AUC** |
| --- | --- | --- | --- | --- | --- | --- |
| RF | 97.73 | 97.22 | 100.00 | 95.45 | 93.33 | 99.03 |
| PC-SVM | 89.61 | 88.89 | 92.86 | 86.36 | 81.25 | 95.45 |
| PC-QDA | 89.61 | 88.89 | 92.86 | 86.36 | 81.25 | 95.13 |
| PC-LDA | 89.61 | 88.89 | 92.86 | 86.36 | 81.25 | 95.13 |
| PLS-SVM | 86.04 | 86.11 | 85.71 | 86.36 | 80.00 | 94.16 |
| PLS-LDA | 86.04 | 86.11 | 85.71 | 86.36 | 80.00 | 94.16 |
| PLS-QDA | 86.04 | 86.11 | 85.71 | 86.36 | 80.00 | 94.16 |

**Table S.4 - Individual Performance Metrics for Tested Models.** This table presents the detailed performance metrics of the models evaluated in the testing phase, including Weighted Balanced Accuracy (WBA), accuracy (ACC), sensitivity, specificity, precision, and recall, which collectively assess each algorithm's effectiveness in detecting hydrocephalus patients.

**
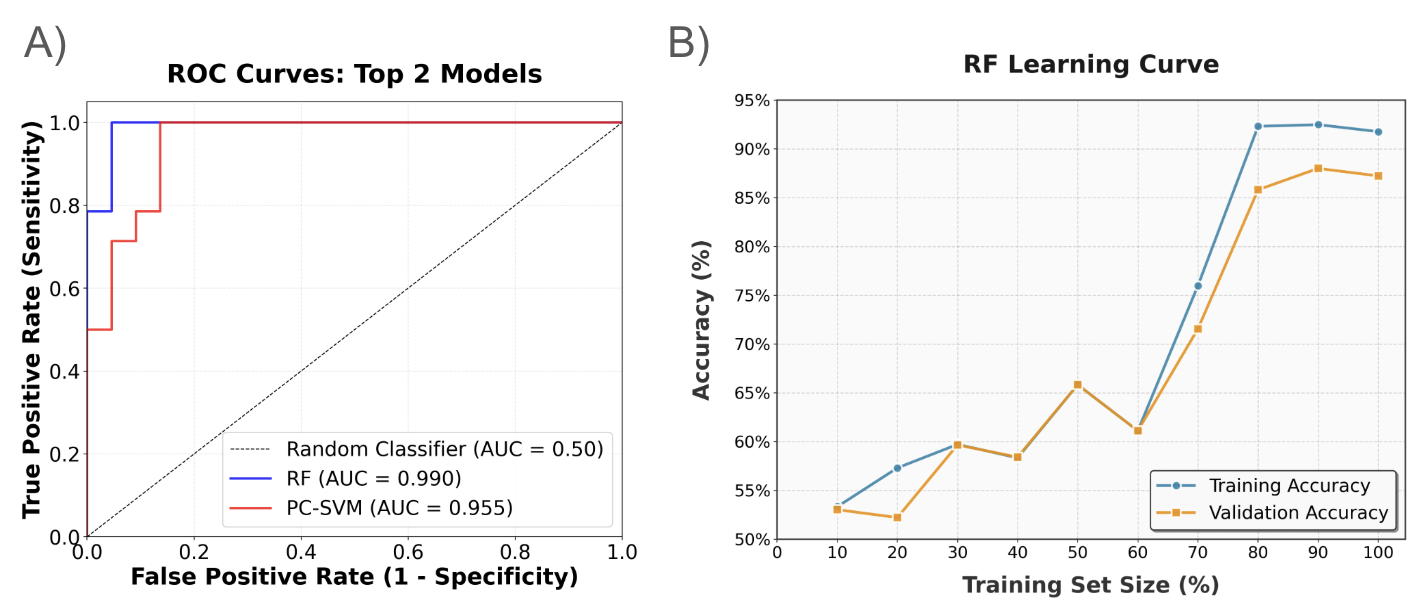
**

**Figure S.4 - Best models performance evaluation using ROC curves and RF learning dynamics.** A) Receiver operating characteristic (ROC) curves for the two best-performing classifiers: Random Forest (RF) and PC-SVM. The RF achieves near-perfect discrimination with an AUC of 0.990, while PC-SVM also performs strongly with an AUC of 0.955; both substantially outperform the random classifier baseline (AUC = 0.50). B) Learning curve for the RF model, showing training and validation accuracy as a function of training set size (10–100%). At small training sizes, the model shows moderate underfitting with lower and unstable validation accuracy. As training size increases, both training and validation performance steadily improve, and closely tracking training accuracy once approximately 70–80% of the data is used. At the largest training sets, the gap between training and validation accuracy narrows, suggesting that the RF generalizes well and benefits substantially from additional training data.

**Supplementary Material 5 – Patient/Sample information.**

| **CSF num.** | **Hospital** | **Ward** | **Age** | **Menin. Diag** | **Hydroc. Diag.** |
| --- | --- | --- | --- | --- | --- |
| 1568 | LCH | M-1 | less 12 years | Negative | Negative |
| 1569 | LCH | M-3 | less 12 years | Negative | Negative |
| 1570 | LCH | NSW | less 12 years | Negative | Negative |
| 1571 | LCH | NSW | less 12 years | Negative | Negative |
| 1596 | LCH | OPD | less 12 years | Negative | Negative |
| 1597 | LCH | E/R | less 12 years | Negative | Negative |
| 1598 | LCH | NEURO | less 12 years | Negative | Negative |
| 1627 | LCH | NEURO | less 12 years | Negative | Negative |
| 1628 | LCH | NSW | less 12 years | Negative | Negative |
| 1647 | LCH | E/R | less 12 years | Negative | Negative |
| 1648 | LCH | E/R | less 12 years | Negative | Negative |
| 1650 | LCH | NSW | less 12 years | Negative | Negative |
| 1651 | LCH | NEURO | less 12 years | Negative | Negative |
| 1679 | LCH | ER | less 12 years | Negative | Negative |
| 1681 | LCH | NSW | less 12 years | Negative | Negative |
| 1682 | LCH | M3 | less 12 years | Negative | Negative |
| 1683 | LCH | DEV | less 12 years | Negative | Negative |
| 1711 | LCH | NNU | less 12 years | Negative | Negative |
| 1712 | LCH | ER | less 12 years | Negative | Negative |
| 1713 | LCH | EMERGENCY | less 12 years | Negative | Negative |
| 1714 | LCH | NEURO | less 12 years | Negative | Negative |
| 1715 | LCH | NSW | less 12 years | Negative | Negative |
| 1717 | LCH | M4 | less 12 years | Negative | Negative |
| 1719 | LCH | M2 | less 12 years | Negative | Negative |
| 1785 | LCH | NSW | less 12 years | Negative | Negative |
| 1792 | LCH | NEURO | less 12 years | Negative | Negative |
| 1816 | LCH | NEURO | less 12 years | Negative | Negative |
| 1818 | LCH | M4 | less 12 years | Negative | Negative |
| 1819 | LCH | ER | less 12 years | Negative | Negative |
| 1849 | LCH | OPD | less 12 years | Negative | Negative |
| 1851 | LCH | OPD | less 12 years | Negative | Negative |
| 1852 | LCH | NEURO | less 12 years | Negative | Negative |
| 1873 | LCH | ER | less 12 years | Negative | Negative |
| 1874 | LCH | M1 | less 12 years | Negative | Negative |
| 1912 | LCH | NSW | less 12 years | Negative | Negative |
| 1913 | LCH | M4 | less 12 years | Negative | Negative |
| 1916 | LCH | NSW | less 12 years | Negative | Negative |
| 1918 | LCH | NNU | less 12 years | Negative | Negative |
| 1919 | LCH | ER | less 12 years | Negative | Negative |
| 1941 | LCH | ER | less 12 years | Negative | Negative |
| 1942 | LCH | NEURO | less 12 years | Negative | Negative |
| 1963 | LCH | NSW | less 12 years | Negative | Negative |
| 1980 | LCH | NEURO | less 12 years | Negative | Negative |
| 2006 | LCH | NSW | less 12 years | Negative | Negative |
| 2008 | LCH | NSW | less 12 years | Negative | Negative |
| 2012 | LCH | NSW | less 12 years | Negative | Negative |
| 2013 | LCH | M3 | less 12 years | Negative | Negative |
| 2014 | LCH | SICU1 | less 12 years | Negative | Negative |
| Lch 3 | LCH | NNU | 23 days | Negative | Negative |
| Lch 4 | LCH | NNU | less 30 days | Negative | Negative |
| Lch 5 | LCH | NNU | less 30 days | Negative | Negative |
| Lch 6 | LCH | NNU | 13 days | Negative | Negative |
| Lch 7 | LCH | NNU | 23 days | Negative | Negative |
| Lch 8 | LCH | NNU | 16 days | Negative | Negative |
| Lch 9 | LCH | NNU | 25 days | Negative | Negative |
| lch 12 | LCH | NNU | less 30 days | Negative | Negative |
| lch 13 | LCH | NNU | 18 dys | Negative | Negative |
| lch 26 | LCH | NNU | 20 days | Negative | Negative |
| lch 27 | LCH | NNU | 8 days | Negative | Negative |
| lch 28 | LCH | NNU | less 30 days | Negative | Negative |
| lch 29 | LCH | NNU | less 30 days | Negative | Negative |
| lch 33 | LCH | NNU | less 30 days | Negative | Negative |
| lch 34 | LCH | NNU | 2 days | Negative | Negative |
| lch 35 | LCH | NNU | 30 days | Negative | Negative |
| lch 37 | LCH | NNU | less 30 days | Negative | Negative |
| lch 40 | LCH | NNU | less 30 days | Negative | Negative |
| lch 46 | LCH | NNU | 10 days | Negative | Negative |
| lch 101 | LCH | NNU | 3 days | Negative | Negative |
| lch 102 | LCH | NNU | 23 days | Negative | Negative |
| lch 103 | LCH | NNU | less 30 days | Negative | Negative |

**Table 1 S.5 – Sample information for Control Samples:** The table provides a detailed summary of cerebrospinal fluid (CSF) samples used as controls for the study. The columns include: CSF num. (unique identifier for each sample), Hospital (all samples collected at Lahore Children’s Hospital), Ward (hospital unit where the patient was admitted, with abbreviations: M for medical ward, NSW for neurosurgical ward, OPD for outpatient department, E/R for emergency room, NEURO for neurology ward, NNU for neonatal unit, DEV for developmental ward, SICU for surgical intensive care unit), Age (due to difficulties in keeping the records from several patients, their age was labelled as less than 12 years), Menin. Diag (meningitis diagnosis, all recorded as "Negative"), and Hydroc. Diag (hydrocephalus diagnosis, all recorded as "Negative"). The controls include diverse patient groups across multiple hospital wards and age ranges, ensuring a representative baseline for comparative analysis.

| **CSF num.** | **Hospital** | **Ward** | **Age** | **Menin. Diag.** | **Hydroc. Diag.** |
| --- | --- | --- | --- | --- | --- |
| CHL0001 | LCH | NNU | 16 days | Negative | Positive |
| CHL0002 | LCH | NNU | 23 days | Negative | Positive |
| CHL0010 | LCH | NNU | 10 days | Negative | Positive |
| CHL0011 | LCH | NNU | 16 days | Negative | Positive |
| CHL0015 | LCH | NNU | less 30 days | Negative | Positive |
| CHL0016 | LCH | NNU | 3 days | Negative | Positive |
| CHL0017 | LCH | NNU | 22 days | Negative | Positive |
| CHL0018 | LCH | NNU | 18 days | Negative | Positive |
| CHL0019 | LCH | NNU | 26 days | Negative | Positive |
| CHL0022 | LCH | NNU | 8 days | Negative | Positive |
| CHL0025 | LCH | NNU | less 30 days | Negative | Positive |
| CHL0036 | LCH | NNU | 5 days | Negative | Positive |
| CHL0041 | LCH | NNU | less 30 days | Negative | Positive |
| CHL0042 | LCH | NNU | 23 Days | Negative | Positive |
| CHL0049 | LCH | NNU | less 30 days | Negative | Positive |
| B1CHL 51 | LCH | NSW | 16 days | Negative | Positive |
| B1CHL111 | LCH | NSW | less 30 days | Negative | Positive |
| B1CHL122 | LCH | NSW | 1 day | Negative | Positive |
| B1CHL 39 | LCH | NSW | less 30 days | Negative | Positive |
| B1CHL 34 | LCH | NSW | less 30 days | Negative | Positive |
| B1CHL112 | LCH | NSW | less 30 days | Negative | Positive |
| B1CHL120 | LCH | NSW | less 30 days | Negative | Positive |
| B1CHL121 | LCH | NSW | less 30 days | Negative | Positive |
| B1CHL40 | LCH | NSW | 16 days | Negative | Positive |
| B1CHL138 | LCH | NSW | 2 months | Negative | Positive |
| B1CHL19 | LCH | NSW | 2.5 months | Negative | Positive |
| B1CHL119 | LCH | NSW | less 30 days | Negative | Positive |
| B1CHL22 | LCH | NSW | less 30 days | Negative | Positive |
| AH 1 | AHCH | NSW | 3 months | Negative | Positive |
| AH 6 | AHCH | NSW | 7 months | Negative | Positive |
| AH35 | AHCH | NSW | 2 months | Negative | Positive |
| AH33 | AHCH | NSW | 13 weeks | Negative | Positive |
| AH 17 | AHCH | NSW | 32 weeks | Negative | Positive |
| AH 28 | AHCH | NSW | 8 weeks | Negative | Positive |
| AH 19 | AHCH | NSW | 23 weeks | Negative | Positive |
| AH 11 | AHCH | NSW | 34 weeks | Negative | Positive |
| AH 4 | AHCH | NSW | 23 weeks | Negative | Positive |
| AH 7 | AHCH | NSW | 38 weeks | Negative | Positive |
| AH 30 | AHCH | NSW | less 12 years | Negative | Positive |
| AH 2 | AHCH | NSW | 28 days | Negative | Positive |
| LGI 33 | LGI | NSW | 1 month | Negative | Positive |
| LGI 26 | LGI | NSW | 6 months | Negative | Positive |
| LGI 16 | LGI | NSW | 3 years | Negative | Positive |
| LGI 14 | LGI | NSW | less 12 years | Negative | Positive |
| LGI 9 | LGI | NSW | 9 months | Negative | Positive |
| LGI 1 | LGI | NSW | 15 weeks | Negative | Positive |
| LGI 11 | LGI | NSW | 4 years | Negative | Positive |

**Table 2 S.5 - Sample information for Hydrocephalus Samples**: This table summarizes cerebrospinal fluid (CSF) samples included in the study as hydrocephalus positives. The columns include: CSF num. (unique identifier for each sample), Hospital (sample collection site: LCH, AHCH, or LGI), Ward (hospital unit where the patient was admitted: NNU for neonatal unit, NSW for neurosurgical ward), Age (patient age, reported in days, weeks, months, or as "less 12 years" where information was not availble), Menin. Diag. (diagnosis for meningitis), and Hydroc. Diag. (diagnosis for hydrocephalus). The dataset represents diverse patient groups, covering neonates, infants, and older children, from multiple hospital wards and institutions.

**Supplementary Material 6 – Morphological characterisation of the AgNP Paper Strips.**


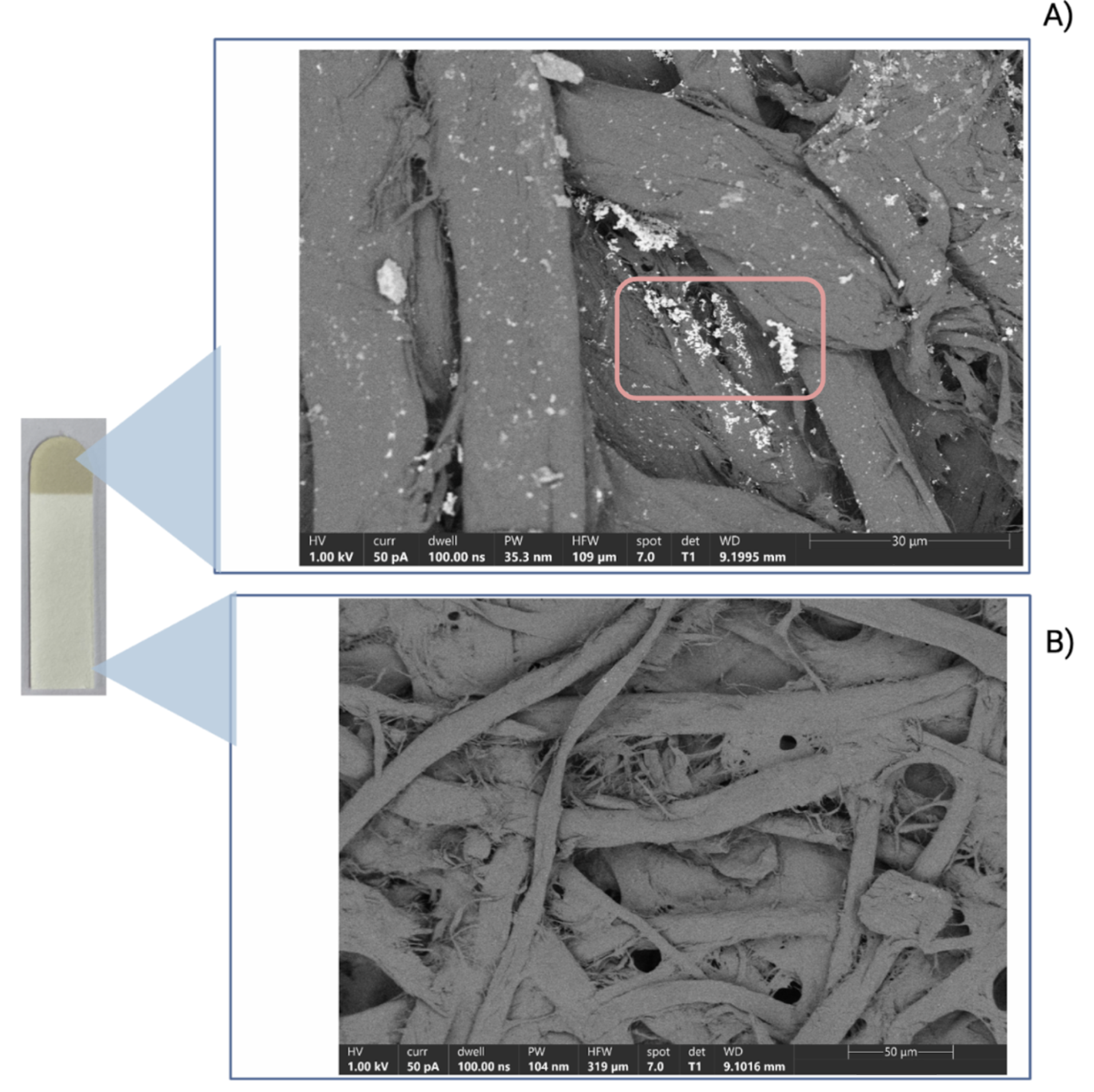

**Figure 1 S.6 – Scanning Electron Microscope (SEM) analysis of AgNp distribution in the cellulose strips.** SEM images of cellulose strips analyzed for AgNP distribution were obtained using a Thermo Fisher Apreo 2S SEM with Optiplan lens mode, a 1 kV accelerating voltage, a 50 pA beam current, a 100 ns dwell time, and a 10 mm working distance. Secondary electrons (topography) were detected using the ETD detector, while backscattered electrons (mass contrast) were captured using the T1 detector. Image (A) is a high-magnification SEM image (scale bar: 30 µm) showing AgNP distribution along the cellulose fibers, with the boxed region highlighting AgNP aggregates. Image (B) is a lower-magnification SEM image (scale bar: 50 µm) depicting the cellulose fiber structure in the absence of AgNPs. The inset on the left illustrates the analyzed cellulose strips, with arrows indicating the corresponding SEM imaging regions.

**
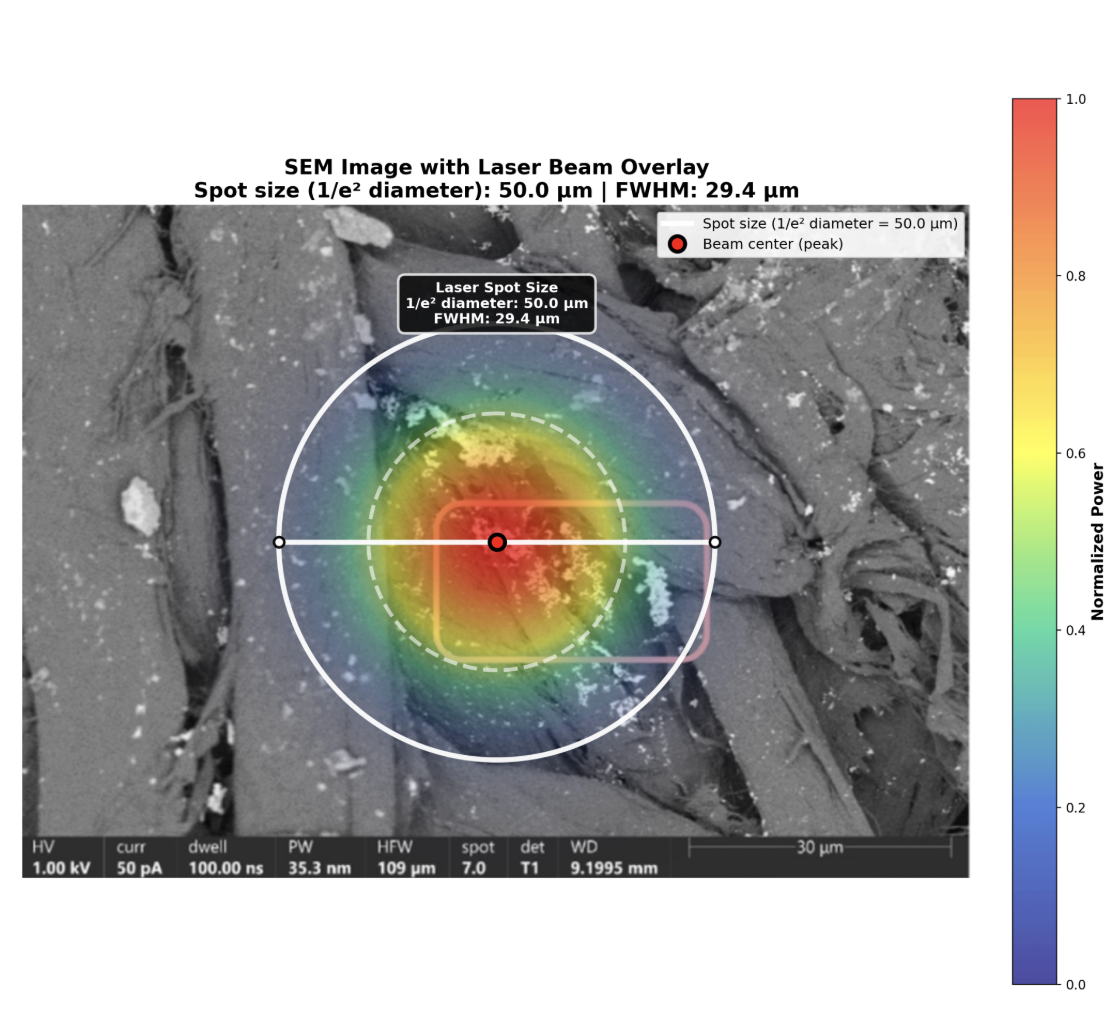
**

**Figure 2 S.6 - SEM image of the Ag-NP strip surface overlaid with the Raman laser beam profile.** The grayscale SEM micrograph shows the nanoparticle-coated cellulose substrate, while the coloured heat map represents the normalized laser power distribution at the focal plane. The white solid circle indicates the measured laser spot size defined by the 1/e21/e^21/e2 diameter (50.0 µm), and the dashed circle marks the full-width at half-maximum (FWHM) region (29.4 µm). The red marker denotes the beam centre corresponding to the peak laser intensity. The visual overlay illustrates the illumination area relative to the substrate microstructure, contextualizing the sampling volume during Raman acquisition.

**SUPLEMENTARY MATERIAL 7 – RAMAN MEASUREMENT SET-UP.**


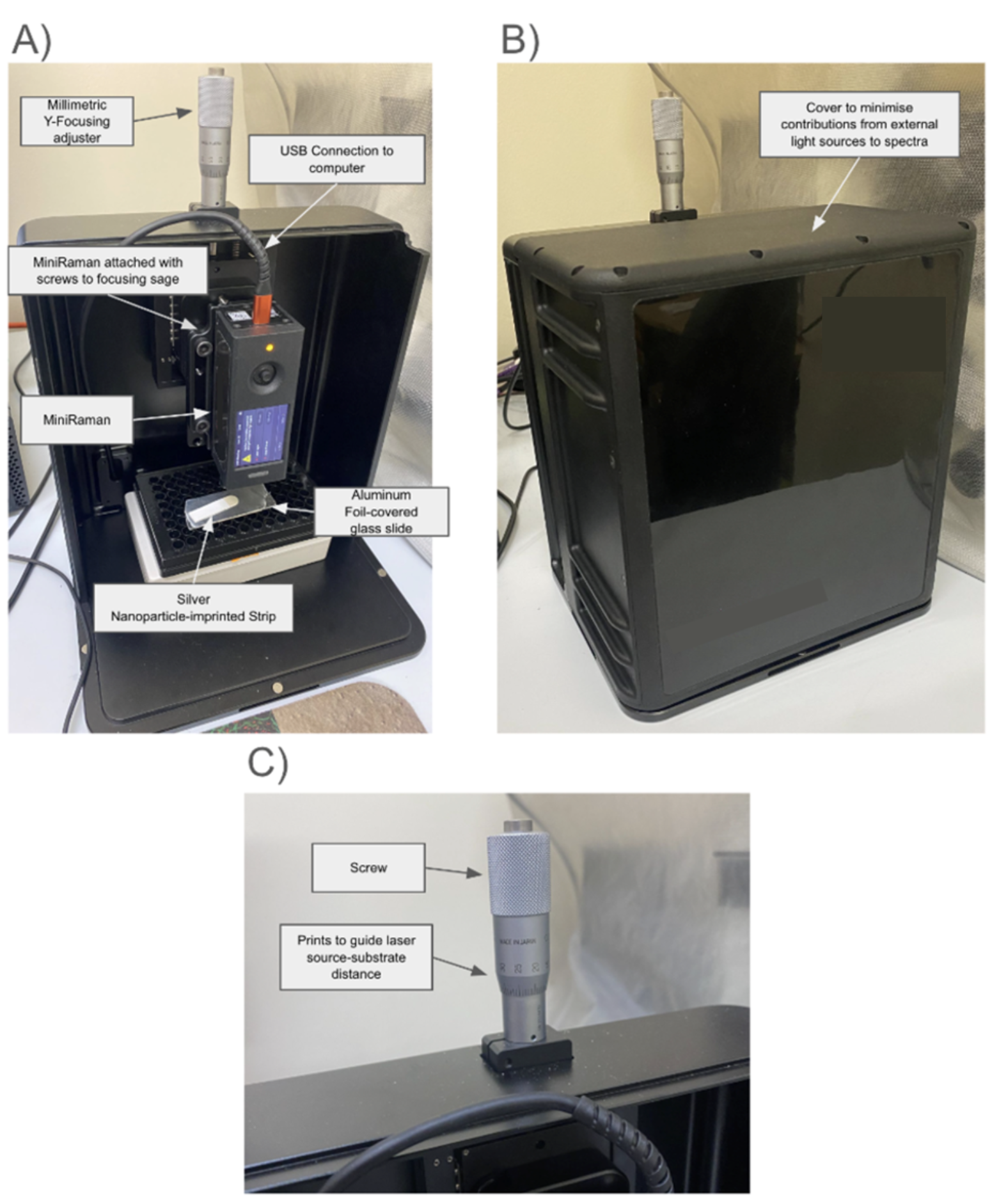


**Figure S.7 - Raman Spectroscopy Measurement Stage Setup: (A)** The experimental setup for Raman measurements using a **MiniRaman spectrometer** mounted on a precise focusing stage. The **Millimetric Y-Focusing Adjuster** enables fine control over the laser-substrate distance, ensuring stable signal acquisition. The silver nanoparticle-imprinted strip is placed on an **aluminum foil-covered glass slide** to enhance the signal. The system is connected to a computer via **USB** for real-time spectral acquisition and control. **(B)** A full view of the **Lightnovo Precise Focusing Stage** with a cover designed to minimize contributions from external light sources, reducing spectral noise and improving signal stability during measurements. **(C)** Close-up of the **Millimetric Y-Focusing Adjuster** screw mechanism with prints to guide the **laser source-substrate distance**. This fine adjustment allows consistent focus and accurate signal acquisition across different measurements. This stage setup was critical for ensuring signal reproducibility and stability during Raman measurements.

**SUPLEMENTARY MATERIAL 8 – VISUALISATION OF THE MODEL SELECTION APPROACH DURING VALIDATION.**


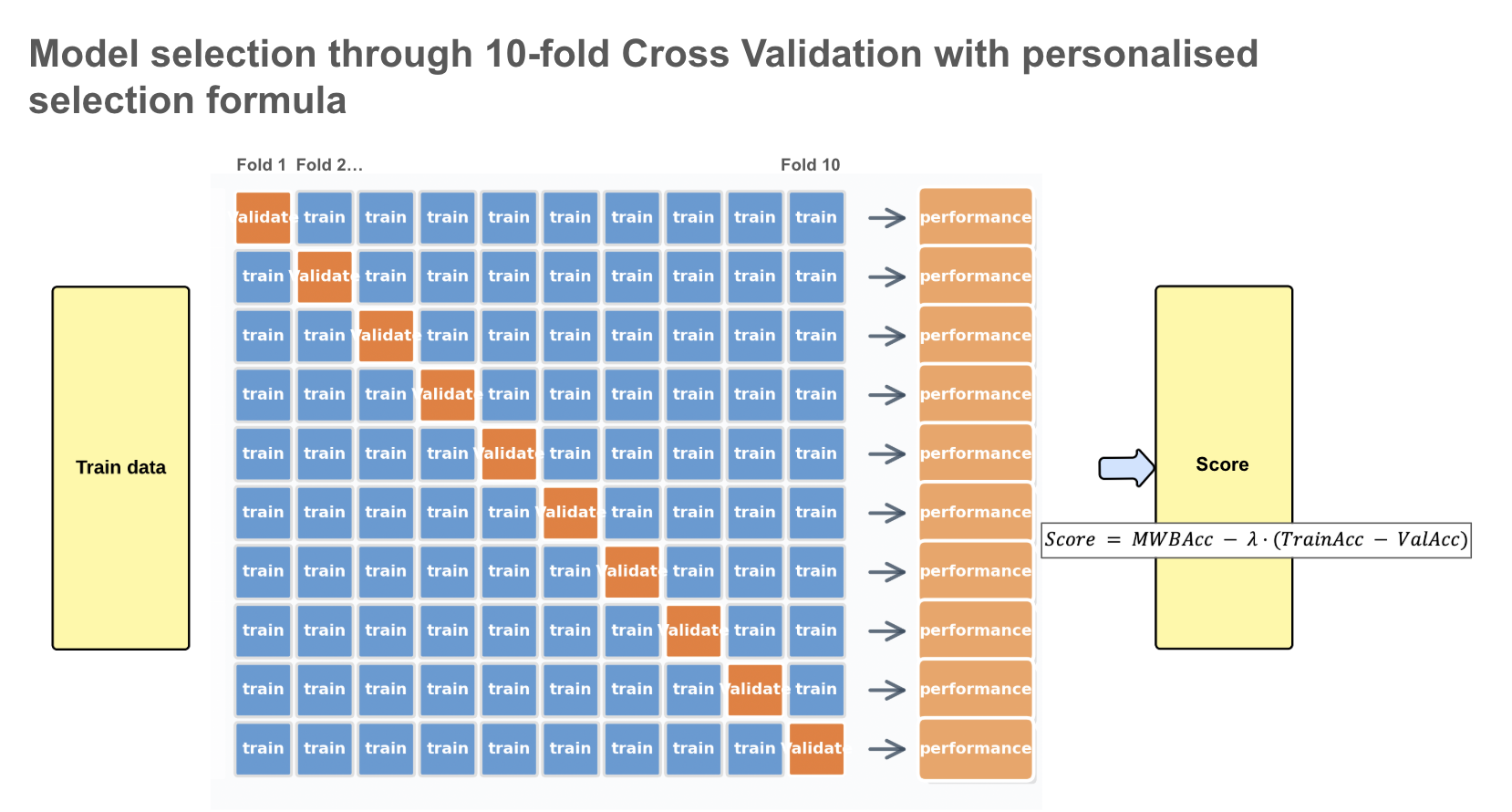


**Figure S.8 - Model selection workflow using 10-fold cross-validation and a customised scoring metric.** Each candidate model is evaluated using 10-fold cross-validation, where in every fold the data are split into a training subset (blue) and a validation subset (orange). For each fold, a weighted balanced accuracy (WBA) is computed using class-inverse weights to ensure that minority classes contribute equally to majority classes. The median of the fold-wise WBA values (MWBA) is then calculated to obtain a robust central accuracy estimate that is resistant to the influence of outlier folds. A final selection score is computed for each model by subtracting an overfitting penalty λ × (TrainAcc – ValAcc) from the MWBA, where the penalty term discourages models whose training accuracy substantially exceeds their validation accuracy. This promotes the selection of models with better generalization behaviour. The hyperparameter combination that yields the highest score is selected as the optimal model. This model is subsequently evaluated on the untouched test set to obtain final performance metrics (accuracy, sensitivity, specificity, precision, and recall).
